# Graphene-Functionalized Titanium Carbide Synthesis and Characterization and Its Cytotoxic Effect on Cancer Cell Lines

**DOI:** 10.7759/cureus.61049

**Published:** 2024-05-25

**Authors:** Devanshi C, Geetha A, Ilangovar IGK, Vasugi S, Balachandran S

**Affiliations:** 1 Department of Physiology, Saveetha Dental College and Hospitals, Saveetha Institute of Medical and Technical Science, Saveetha University, Chennai, IND

**Keywords:** mtt assay, xrd, sem, cancer cells, cytotoxic effect, graphene, mxene

## Abstract

Background

Graphene is a versatile material with promising applications in various fields such as electronics, energy, biomedicine, and the environment due to its exceptional mechanical strength, thermal and electrical conductivity, transparency, and chemical stability. Graphene has been extensively used in biological and medical settings. MXene is a two-dimensional (2D) material that exhibits a strong affinity for water and electrical conductivity because of its surface terminations (oxygen {-O}, fluorine {-F}, and hydroxyl {-OH}) and transition metal carbide or nitride. MXene has attracted significant attention recently for its wide range of applications and unique properties. This study focuses on the synthesis and characterization of graphene-functionalized MXene. Furthermore, we investigated its cytotoxic effects on cancer cell lines. The characterization of graphene-functionalized MXene is carried out using scanning electron microscopy (SEM), X-ray diffraction(XRD), and Fourier transform infrared spectroscopy (FTIR) assays.

Materials and methods

Graphene powder was finely ground in isopropyl alcohol and then sonicated for two hours to produce solution A. MXene was synthesized by reacting titanium aluminum carbide (Ti_3_AlC_3_) with hydrofluoric acid (HF). A mixture of Ti_3_AlC_3_ and HF was heated to 40°C with continuous stirring for 24 hours to form solution B. Subsequently, solutions A and B were combined and stirred for 30 minutes. The resulting mixture was transferred to a hydrothermal reactor and maintained at 180°C for 12 hours. After the completion of the reaction, the resulting material was cooled to room temperature and purified through washing with distilled water, ethanol, and acetone. The sample was then dried at 80°C for 12 hours.

Results

The X-ray diffraction (XRD) study confirms the formation of graphene-functionalized titanium carbide (Ti_3_C_2_). The sharp peaks indicate a highly crystalline nature. Graphene is a sheet-like structure with numerous gaps. Particles exhibit a multitude of voids and pores on their surfaces. Upon incorporation, graphene displays a small sheet-like structure. Graphene-functionalized titanium carbide confirms the presence of distinct layered or sheet-like structures stacked together. Following the addition of the material, some cancer cells are eradicated, and they exhibit increased biocompatibility, demonstrating anticancer activity.

Conclusion

Graphene-functionalized titanium carbide has been successfully synthesized and characterized, as evidenced by various analytical methods such as X-ray diffraction (XRD), scanning electron microscopy (SEM), and methyl-thiazoldiphenyl-tetrazolium (MTT) assays. The cytotoxic impact of the synthesized graphene-functionalized titanium carbide on cancer cell lines was examined. The findings reveal a notable cytotoxic effect, indicating its potential as an anticancer agent. Further research in collaboration with experts from diverse fields will be crucial to advance and translate this technology into practical applications for cancer patients.

Future scope

Graphene and titanium carbide are promising materials for cancer research, biomedical applications, and imaging. Nevertheless, additional research is required to comprehend their mechanisms, enhance their properties, assess their safety and efficacy, and conduct clinical trials.

## Introduction

Graphene, a two-dimensional (2D) carbon (C) allotrope, comprises a single layer of carbon atoms. The term "graphene" is derived from graphite, another carbon allotrope with multiple double bonds. Graphene's structure consists of 2D carbon atoms arranged in a hexagonal pattern connected by sp^2^ hybrid bonds. The carbon atoms are arranged in a honeycomb pattern to form graphene, showcasing one pi (π) orbital and three sigma (σ) bonds extending perpendicularly to the plane. The perpendicular π bonds govern the interactions between adjacent graphene layers, while the σ bonds maintain the hexagonal structure. Changes in graphene's chemical composition often arise from sp^3^ hybridization or the absence of sp^2^ carbon atoms. Graphene's electrical structure comprises six π orbitals, classifying it as a semimetal. The band gap separates these six orbitals into three bonding and three antibonding orbitals. Due to the fusion of benzene rings, the valence and conduction bands are closely packed, allowing electrons to transition freely without generating heat. Research frequently utilizes graphene oxide (GO) and reduced graphene oxide (rGO) as two variations of graphene. The unique properties of graphene stem from the covalent bonding of carbon atoms within the plane, forming σ bonds with three neighboring carbon atoms and one out-of-plane π bond. Graphene's exceptional electrical conductivity and strength set it apart from other materials. Its remarkable physicochemical characteristics include mechanical strength, high electron mobility at room temperature, thermal conductivity, electrical conductivity, stiffness, extensive surface area, and impermeability to gases. The malleable nature of graphene enables it to adopt various forms, such as spheres, tubes, and stacked graphite structures [1,2].

MXene is a two-dimensional molecular sheet. A common method for producing MXene involves removing the A layers from MAX phases through etching. The molecular formula for these MAX phases is M_n+1_AX_n_, where M represents a transitional metal, n is a number between 1 and 3, A is an element from group IIIA or IVA (such as silicon, gallium, or aluminum {Al}), and X can be carbon, nitrogen, or a combination of both. These phases are ternary carbides or nitrides. The hexagonal MAX phases consist of densely packed M layers and X-occupied octahedral sites, with A atoms connecting the M and X layers. In MAX phases, the M-X bond exhibits a combination of ionic, metallic, and covalent properties, while the M-A bond typically displays a metallic nature. Unlike graphite, where layers are held together by weak van der Waals forces, the strong interlayer bonding in MAX phases prevents the mechanical separation or breakage of these connections. The M-X bonds are significantly stronger than the M-A bonds, enabling the selective chemical etching of the A layers without affecting the M-X links. The resulting weakly bound layers of M_n+1_X_n_ can be isolated simply through sonication. These two-dimensional materials are known as MXene, emphasizing the removal of A layers from the original MAX phase, and share similarities with graphene in their two-dimensional nature. As the M_n+1_X_n_ units are etched, functional groups such as oxygen (-O), hydroxyl (-OH), and fluorine (-F) continuously adhere to their surfaces. These surface functional groups are represented as T_x_ in the chemical formula of MXene, denoted as M_n+1_X_n_T_x_. The proportions of surface functional groups on MXene vary depending on the etching conditions, presenting a mystery [3-5].

Due to the presence of surface terminations (-O, -F, and -OH) and transition metal carbide/nitride, MXene exhibits a unique combination of heightened hydrophilicity and metallic conductivity. With exceptional physicochemical properties, such as excellent electrical conductivity, large surface areas, optical and magnetic capabilities, and remarkable thermal and mechanical characteristics, MXene shows significant potential for future biological applications. The exceptional flexibility of MXene, coupled with its two-dimensional morphology and layered structures, facilitates seamless interactions with other materials. MXene possesses both biocompatibility and biodegradability, allowing for efficient elimination from the human body. The notable advancements of MXene in the field of nanomedicine for cancer treatment, bioimaging, biosensors, and antibacterial agents may be attributed to these attributes. The integration of MXene with graphene in a hybrid/composite configuration can address various unmet needs across different industries, particularly in the fields of medicine and biomedical engineering [5,6]. MXene demonstrates considerable promise for effective and minimally invasive anticancer treatment due to its distinct photothermal, chemotherapeutic, and photodynamic properties. Researchers have explored its potential for the precise delivery of anticancer medications and therapies, utilizing heat and light for cancer treatment. The antitumor effectiveness of the graphene platform was enhanced through chemical functionalization. Graphene-based combination chemotherapy and phototherapy offer the ability to target cancer cells specifically [7].

Graphene and MXene are utilized for their potential as photodynamic therapeutic agents. Cancer is one of the leading causes of death globally, making it essential for cancer research to innovate new advanced and targeted methods for early detection and treatment to improve diagnosis outcomes and reduce treatment side effects [8]. Graphene and MXene belong to the 2D material group and are extensively studied as multimodal nanoplatforms for cancer diagnosis and treatment, specifically leveraging their potential as photodynamic therapeutic agents. Due to their unique physicochemical properties, they are valuable assets for photodynamic therapy (PDT) in conjunction with bioimaging, photothermal therapy, drug delivery, and gene delivery [9]. Cytotoxicity assessments, encompassing both in vitro and in vivo techniques, are commonly used to explore material toxicity, evaluate cell viability, and assess tissue irritations. The methyl-thiazoldiphenyl-tetrazolium (MTT) test gauges the mitochondrial activity of viable cells to determine cell viability and evaluate the cytotoxic impact of a drug in an in vitro environment. In vitro cytotoxicity assays are considered more reliable for evaluation due to their simplicity, cost-effectiveness, and reproducibility [10].

The reasons why previous studies failed to reach their intended conclusions are as follows: firstly, results from the visible light spectrum indicate that these tools are only effective for treating surface-level tumors such as skin cancer. Secondly, the long-term safety of these 2D materials needs to be thoroughly examined to understand their potential risks before being used in clinical settings. Schottky junctions are often used to create an internal electrical field, which is known to be a useful tactic for enhancing carrier separation. Excellent antibacterial action is shown by titanium carbide (Ti_3_C_2_) nanosheets, which in less than three hours dramatically kill bacterial cells and cause cell DNA release and dispersion. Thus, for photocatalytic bacteriostatic applications, a semiconductor having an antibacterial action combined with MXene may be used. Lastly, it is essential for all projects utilizing synthetic nanomaterials to ensure comprehensive characterization and reproducibility to prevent overestimating their therapeutic capabilities. The utilization of graphene-functionalized MXene in cancer cell lines was essential due to the lack of in-depth studies on potential non-targeted toxicity, detailed characterization, biodegradability, and specific physical-chemical properties before evaluating them for cancer treatment. Therefore, this study addresses these research gaps. Additionally, it aims to explore the potential of MXene and graphene for cancer theranostics, enhance understanding of nanoparticle interactions with tumors, establish regulatory protocols for personalized therapy approaches, and investigate the toxicity of MXene. Consequently, this research seeks to assess the cytotoxic impact of graphene-functionalized MXene on cancer cell lines. The synthesis process is analyzed using various techniques, such as X-ray diffraction (XRD), scanning electron microscopy (SEM), and MTT assay studies.

## Materials and methods

Preparation of a graphene solution

Two grams of potassium persulfate was added to 25 g of sulfuric acid in a container and mixed well in a small beaker. The solution was stirred after adding 0.5 g of graphite to ensure a homogeneous suspension. After transferring the acidic mixture to a 100 mL round-bottom flask, it was placed in a silicon oil bath and stirred magnetically at 150 revolutions per minute (rpm) while kept at 70°C. After five minutes, the mixture began to show many bubbles. The chemical expansion (potential exfoliation) of the graphite probably caused the magnetic stirrer to cease revolving after another 10 minutes. Afterward, the flask was taken out of the oil bath, and 100 mL of water was added gradually to stop the reaction, which caused the mixture to expand and exfoliate even more. We observed that the temperature of the mixture rose quickly to about 120°C. The next step was to filter the mixture and then wash it thoroughly with acetone to get rid of any remaining acids. Feathered graphene platelets were obtained by drying the product in a fanned oven at 60°C for two hours. Graphene nanosheets (50 mg) are thoroughly ground with a pestle. This process aids in breaking down any clusters and ensures a more even distribution throughout the solvent. Pour the isopropyl alcohol (30 mL) into a suitable container; then, add the ground graphene nanosheet to the isopropyl alcohol. Gently stir the mixture to ensure proper blending. Place the container (solution A) in an ultrasonicator for two hours. This solution contains uniformly dispersed graphene nanosheets in the solvent, along with any remaining reactants and by-products.

Preparation of monolayer Ti_3_C_2_ MXene nanosheets

Titanium aluminum carbide (Ti_3_AlC_2_) is utilized as the initial material in producing titanium carbide via a chemical process. The Ti_3_AlC_2_ is added with a 20 mL solution consisting of a 9 M concentration of hydrochloric acid (HCl) and 3 g of lithium fluoride (LiF_3_). This mixture acts as an etchant, targeting the aluminum layers on Ti_3_AlC_2_ while safeguarding the desired MXene layers. The Ti_3_AlC_2_ and hydrofluoric acid (HF) mixture is subjected to a temperature of 40°C. Applying heat to the mixture is expected to expedite the etching process, facilitating the removal of the aluminum layers. Furthermore, continuous stirring ensures the uniform dispersion of the substances participating in the reaction and enhances the efficacy of the etching process. Following 24 hours of heating and stirring, the reaction produces a substance known as solution B. This solution contains the resulting MXene flakes evenly dispersed in the solvent, along with any remaining initial components and by-products from the reaction. The unreactant was washed with water and ethanol. The final product was vacuum-dried at 80°C for 12 hours.

Synthesis of graphene-functionalized Ti_3_C_2_ MXene sheets

The dispersion of graphene in solution A is gradually introduced into solution B, containing Ti_3_C_2_ MXene while stirring continuously for 30 minutes. The MXene-graphene suspension undergoes hydrothermal treatment in a sealed Teflon-lined stainless steel hydrothermal reactor. This specialized container is used for conducting chemical reactions in an aqueous medium under high temperatures and pressures. Subsequently, the hydrothermal reactor is placed in an oven, and the reaction is carried out at elevated temperatures (180°C) for 12 hours. This process facilitates the integration of graphene-functionalized MXene nanosheet matrices. Upon the completion of the reaction, the material is removed and allowed to cool to room temperature. The synthesized material is purified by sequentially passing it through distilled water, ethanol, and acetone. Finally, the material is dried for 12 hours at 80°C. Following the preparation, the material was characterized using various analytical techniques [11].

Cell line and culture of SW480

The SW480 cell line (human colorectal adenocarcinoma) was obtained from the National Centre for Cell Science (NCCS) in Pune, India. SW480 cells were grown in Dulbecco's Modified Eagle Medium (DMEM) growth media supplemented with 10% fetal bovine serum (FBS) and 1% penicillin-streptomycin antibiotic (Sigma-Aldrich). The cells were maintained at 37°C in an incubator with 5% carbon dioxide (CO_2_) and 95% air. Dimethyl sulfoxide (DMSO) was used as a control at a concentration of less than 0.2%.

MTT assay

SW480 cells, at a concentration of 1 × 10^4^ cells, were seeded on a 96-well plate and incubated overnight to support their growth. After incubation, the cells were exposed to 50 µg/mL of graphene-functionalized MXene. Subsequently, the cells were placed in a CO_2_ incubator at 37°C with 5% humidity for 24 hours. Following this incubation period, the growth medium was aspirated, and 25 μL of a phosphate-buffered saline (PBS) solution containing 0.25 mg mL^-1^ of MTT reagent was added to each well. The culture, along with the MTT reagent, was then incubated at 37°C with 5% CO_2_ for an additional 24 hours. After removing the medium with the MTT reagent, the insoluble formazan crystals were dissolved in DMSO. The absorbance at 570 nm for each well was measured using a spectrophotometer [12].

## Results

X-ray diffraction (XRD)

**Figure 1:**
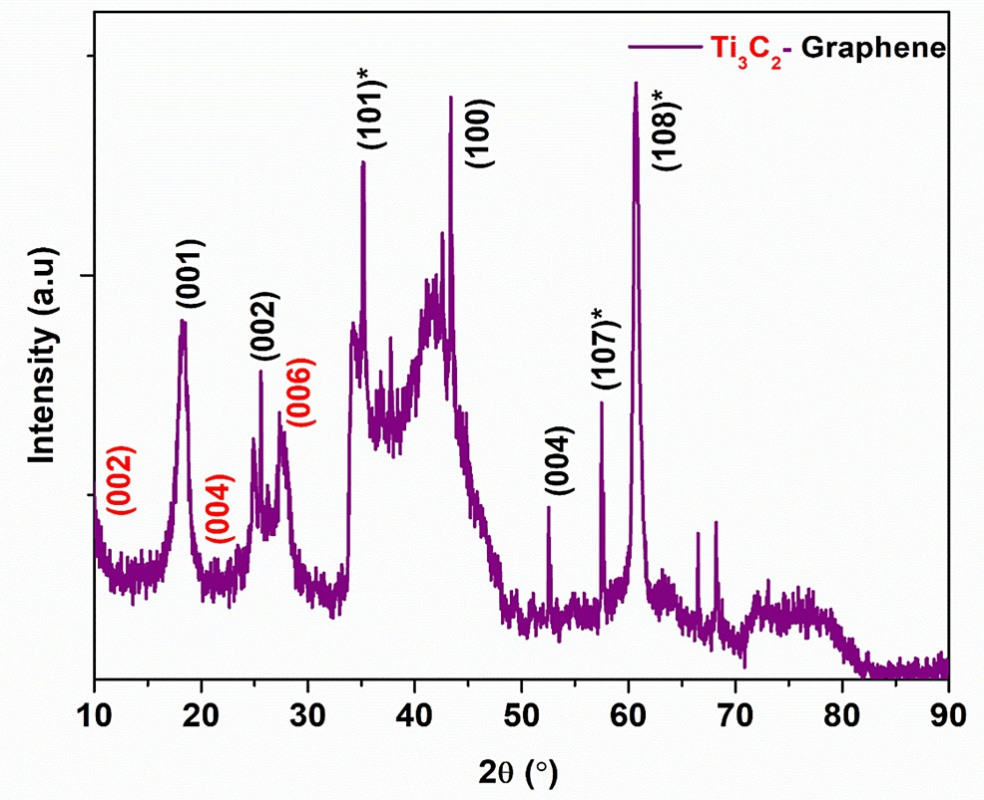
XRD analysis of Ti3C2-graphene *High crystalline primary peaks XRD, X-ray diffraction; Ti_3_C_2_, titanium carbide

XRD spectra of graphene-functionalized MXene (Ti_3_C_2_-graphene) were measured within a range of 2θ from 10° to 90°. The main peaks of MAX are observed at 19.1°, corresponding to the (004) crystallographic planes. However, the aluminum etching process has shifted these peaks toward lower angles, specifically at 18.62°, corresponding to the (004) planes. This shift toward a lower angle indicates an increase in the interplanar distance. The peaks at 2θ = 18.62°, 36.28°, 43.28°, 60.44°, and 72.80° correspond to the (004), (111), (200), (220), and (311) lattice planes of Ti_3_C_2_ MXene, respectively. The sample shows (002) diffraction peaks at 25.6° and another peak at 45°, indicating the presence of a graphene structure. Figure 1 shows the main diffraction peaks of Ti_3_C_2_-graphene, which shows that the Ti_3_C_2_-graphene is arranged in crystals. The XRD analysis reveals that the peaks exhibit broadening and reduced intensity due to the presence of etched MXene sheets. The detection of all the main peaks of graphene and Ti_3_C_2_ MXene provides conclusive evidence of the successful synthesis of Ti_3_C_2_-graphene. A new weak diffraction peak of the (006) lattice plane appears at 27.6. Meanwhile, the characteristic peak of (104) lattice planes of Ti_3_AlC_2_ disappears, which indicates that the interlayer of Al atoms in Ti_3_AlC_2_ is efficiently removed and Ti_3_AlC_2_ has been successfully transformed into Ti_3_C_2_. The inclusion of graphene also reduced the usual layer spacings of Ti_3_C_2_ sheets.

Field emission and scanning electron microscopy (FE and SEM)

**Figure 2:**
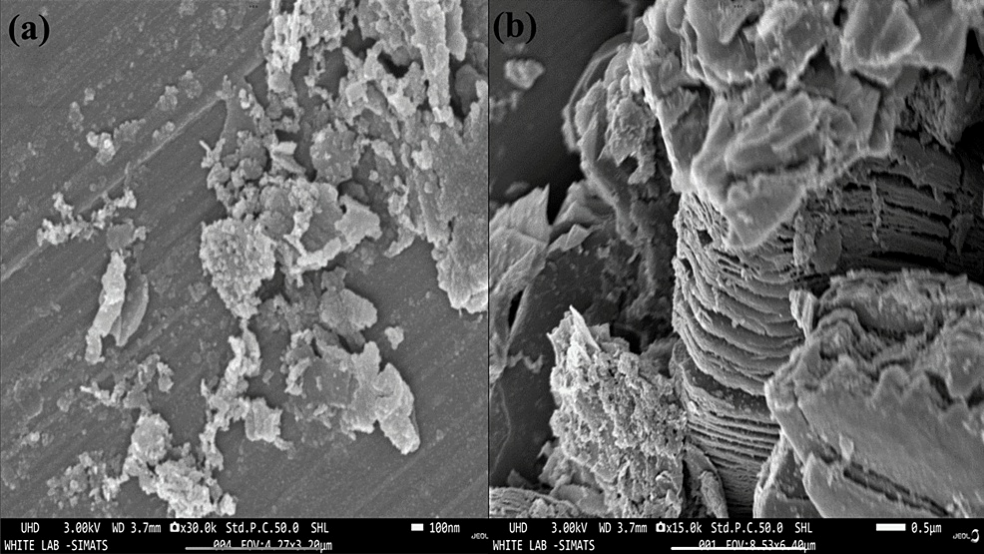
SEM analysis of Ti3C2-graphene at different places: (a) 100 nm and (b) 0.5 µm SEM, scanning electron microscopy; Ti_3_C_2_, titanium carbide

The FE and SEM images of Ti_3_C_2_-graphene were captured using an ultra-high-definition (UHD) microscope operating at 3.00 kV with a working distance (WD) of 3.7 mm. The layered structure of the nanosheets during the etching process of Ti_3_C_2_ MXene was observed through field emission and scanning electron microscopy (FE and SEM) images of Ti_3_C_2_-graphene. The figure demonstrates the successful etching of the Ti_3_C_2_ from its MAX phase by eliminating the Al layer, as indicated by the presence of numerous nanoflakes. Figure 2b shows that the nanosheets produced are extremely thin and consist of multiple layers, displaying clear and well-defined boundaries. This confirms the high quality of the manufacturing process. The efficient separation of Ti_3_C_2_-graphene layers was confirmed by field emission and scanning electron microscopy (FE and SEM). Ti_3_C_2_ possesses a sheet-like structure with various gaps. When combined, graphene adopts a dense, sheet-like structure. Figure 2b depicts the surface morphology of the Ti_3_C_2_-graphene, validating the existence of distinct layered or sheet-like structures arranged in an accordion-like manner. The FE and SEM images indicated that the synthesized Ti_3_C_2_-graphene exhibits a highly crystalline morphology. The presence of Ti_3_C_2_-graphene results in increased roughness on the surface. The heterostructure with such a distribution of Ti_3_C_2_-graphene not only improves space utilization but also effectively prevents the self-restacking of graphene sheets.

Energy-dispersive X-ray spectroscopy (EDAX) analysis

**Figure 3:**
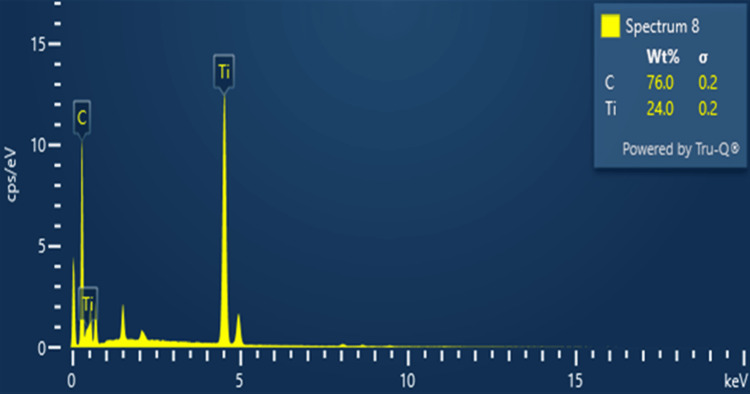
EDAX analysis of graphene-functionalized Ti3C2 nanosheets EDAX, energy-dispersive X-ray spectroscopy; Ti_3_C_2_, titanium carbide; C, carbon; Ti, titanium; σ, sigma; Wt%, weight percentage

The EDAX analysis proves the existence of titanium (Ti) and carbon (C) in these prepared materials. EDAX analysis can detect the elemental composition of Ti_3_C_2_-graphene that arises from the surface functionalization of the material. The weight percentages of carbon and titanium are 76.0% and 24.0%, respectively. The discussion explains integrating graphene as dopants and their possible impact on material characteristics, such as heightened conductivity or increased chemical reactivity. The EDAX analysis confirms the purity of prepared Ti_3_C_2_-graphene materials; no other impurity is present (Figure 3).

MTT assay study of membrane and SW480 cell lines

**Figure 4:**
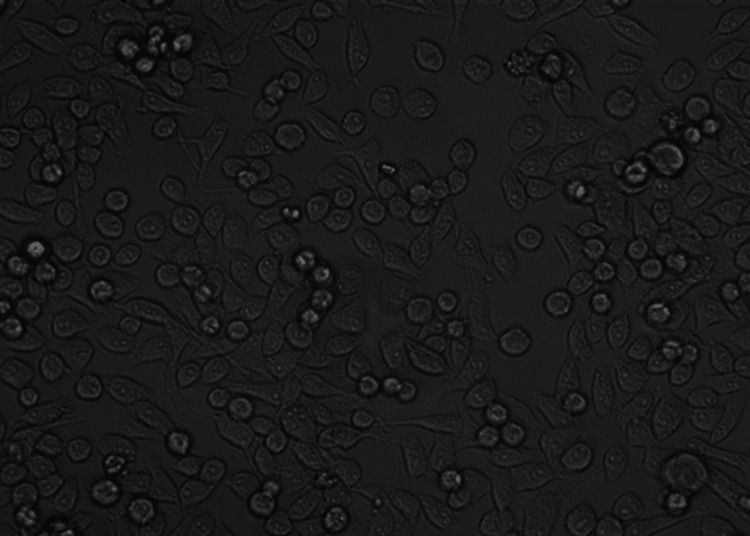
MTT assay study of graphene-functionalized Ti3C2 nanosheets and cancer cell line (after 48 hours) MTT assay: 3-(4,5-dimethylthiazol-2-yl)-2,5-diphenyl tetrazolium bromide assay Ti_3_C_2_, titanium carbide; MTT, methyl-thiazoldiphenyl-tetrazolium

The MTT assay is a widely used colorimetric method to assess cell viability and proliferation, commonly applied in anticancer research to evaluate the cytotoxic effects of compounds on cancer cells. This assay focuses on the enzymatic reduction of MTT (3-(4,5-dimethylthiazol-2-yl)-2,5-diphenyl tetrazolium bromide) by mitochondrial dehydrogenase, leading to the formation of a purple formazan product. The quantity of formazan generated is directly linked to the number of viable cells. In this study, we investigated the influence of Ti_3_C_2_-graphene on SW480 cells and observed its ability to inhibit cell proliferation. The MTT assay results confirmed a decrease in cell numbers following the introduction of Ti_3_C_2_-graphene, supporting its anticancer properties. Our findings indicated that Ti_3_C_2_-graphene exhibited cytotoxic effects (Figure 4).

## Discussion

In a study by Scheibe et al., a similar MXene membrane was prepared, and its cytotoxic effect on both tumorigenic and nontumorigenic cell lines was studied in vitro. We examined the cytotoxic effects of various Ti_3_C_2_T_x_ MXene structures and their precursors (TiC, Ti_2_AlC, and Ti_3_AlC_2_) on human fibroblast and cervical cancer cells. The findings indicated that exposure to higher amounts of particles smaller than 44 μm, such as those in the TiC and MAX phases, could be harmful as they might harm the cell membrane. MXene structures demonstrated excellent compatibility with cells, with cell viability exceeding 80% at all tested doses after 48 hours of exposure. Therefore, it is essential to be cautious when removing these impurities from multilayered MXene samples. The concentration led to a unique cytotoxic response specific to the cell line. Additionally, there was an increase in reactive oxygen species (ROS) formation and mechanical stress. These outcomes stemmed from the inherent metabolic differences between cancerous and noncancerous cells. Even at the highest concentrations, the Ti_3_C_2_T_x_ forms displayed minimal cytotoxicity toward MSU 1.1 cells. The cytotoxic behavior also varied depending on the cell type, with greater cytotoxicity observed in cells of cancerous origin [13]. Zhang et al.'s study highlighted the biological influence of MXene on living cells by inducing ROS production. Cancer cells typically have elevated ROS levels due to increased anabolic and catabolic activities. MXene shows promise as a potential anticancer agent [14].

An extensive investigation was conducted to analyze the biological effects of GO nanosheets. The study specifically focused on the impact of fetal bovine serum (FBS), a commonly used component in cell culture medium, on the toxicity of graphene oxide (GO). Human cells exhibited varying levels of cytotoxicity in response to the presence of GO at a low FBS concentration (1%). However, the detrimental effects of GO were significantly reduced when the FBS concentration was increased to 10%, which is the standard in cell medium. The research suggests that the cytotoxicity of GO nanosheets is a result of direct interactions with the cell membrane, leading to physical damage. This effect is notably diminished when GO is exposed to FBS due to its high affinity for protein binding. GO has been found to induce cytotoxicity in human alveolar basal epithelial cells, with toxicity levels varying based on the dosage [15]. The chemical modification of graphene oxide by introducing sulfonic acid groups enhances its stability in physiological solutions. Subsequent bonding of folic acid (FA) molecules to graphene oxide enables specific targeting of Michigan Cancer Foundation-7 (MCF-7) cells and human breast cancer cells with FA receptors. The study explores the loading of two anticancer drugs, doxorubicin (DOX) and camptothecin (CPT), onto FA-conjugated GO (FA-GO) through π-π stacking and hydrophobic interactions. Research indicates that FA-GO carrying both anticancer drugs exhibits targeted delivery to MCF-7 cells and significantly higher toxicity compared to GO carrying only DOX or CPT [16].

Cellular uptake, apoptosis assessment, and cellular morphology analysis at different doses indicated a significant toxic effect of GO-zinc oxide (ZnO) on MCF-7 cells. Additionally, GO-ZnO was found to be cytotoxic at higher doses (60-100 µg/mL). The optimal incubation period was determined to be 24 hours, during which GO-ZnO effectively targeted the cells. The MCF-7 cellular model showed a dose-dependent decrease in cell viability. Evidence suggests that GO-ZnO nanocomposites induce cytotoxicity and oxidative stress in MCF-7 cells, as evidenced by the production of reactive oxygen species (ROS) and the lactate dehydrogenase (LDH) test [17,18]. Cell viability decreased significantly, reaching 63%, indicating that ROS generation plays a crucial role in causing extensive damage to cancer cells. This damage occurs through necrosis or apoptosis, initiated by oxidative stress. Within 24 hours, MCF-7 cells exhibited an apoptotic response characterized by nuclear margination. GO-ZnO nanocomposites show promise for biological applications [19]. Another study reported that the addition of cobalt oxide (Co_3_O_4_) nanoparticles to GO enhances its anticancer properties, resulting in a nanocomposite with potent anticancer effects. The Co_3_O_4_/GO nanocomposite synergistically prevents cell aggregation and induces cytotoxicity by generating free radicals through Co_3_O_4_, leading to cancer cell eradication [19,20]. An experiment demonstrated that exposing cancer cells to 200 µg/mL of GO resulted in a cell viability of 60%. However, when cancer cells were exposed to a compound of cobalt oxide and graphene, their cell viability decreased to 37%, indicating stronger anticancer properties of the cobalt oxide and graphene combination [20-22].

Graphene-based nanocomposites have improved capabilities in bioimaging, cancer detection, and therapy. Our current understanding of how cells absorb these compounds is insufficient, particularly regarding the amount taken in by the cells. This knowledge is crucial for determining their functions and ensuring their safety. Researchers developed covalently bonded GO/gold (Au) and GO/silver (Ag) composites by incorporating Au and Ag nanoparticles into previously disulfide bond-treated GO sheets. The quantification of the cellular internalization of these composites was carried out using an ion beam microscope (IBM) to evaluate the metal content within human lung cancer cells (A549 cells) and liver hepatocellular carcinoma cells (HepG2 cells). Cell uptake investigation was performed using inductively coupled plasma mass spectrometry (ICP-MS), a highly sensitive technique commonly utilized for analyzing cell suspensions. The study also explored the toxicity resulting from the cellular uptake of GO-derived composites. The potential cause of harm was also hypothesized based on the measurement of nanoparticles within the cells. The physical characteristics of graphene, its interactions with cells, and its accumulation in specific organs largely dictate its potential adverse effects. Graphene can produce reactive oxygen species when exposed to light. This may lead to oxidative stress, cellular dysfunction, pro-inflammatory reactions, and mitochondrial damage. Graphene penetration into the nucleus can cause genetic harm and cell death by disrupting DNA strands, activating transcription factors for gene expression, and triggering cell apoptosis. The nanosheets of the rGO-Ag nanocomposite exhibited remarkable solubility and durability, lasting over three months. The rGO-Ag nanocomposite showed significant cytotoxicity and demonstrated highly effective apoptotic effects on ovarian cancer cells. The nanomaterials utilized in our study had a synergistic impact, indicating that the combined approach was more potent than the individual effectiveness of each nanomaterial. This unique formulation holds promise for enhancing the development of more potent anticancer drugs and could offer innovative cancer therapies, particularly targeting cancer stem cells (CSCs) [23,24].

Limitation

Synthesis variability poses a challenge, potentially resulting in inconsistent material properties. Analyzing graphene-functionalized titanium carbide entails the complexity of using sophisticated techniques with inherent limitations. Contaminants affecting material purity could impact cytotoxicity results if not adequately addressed. In vitro assays may not fully replicate in vivo conditions or encompass diverse cancer types. Biocompatibility and toxicity considerations are crucial due to potential issues. The understanding of cellular uptake and mechanisms of action remains challenging. Conducting in vivo studies is essential but resource-intensive. These tasks necessitate meticulous experimental planning, interdisciplinary collaboration, and a comprehensive grasp of both material science and biological systems.

## Conclusions

The present investigation focuses on the cytotoxic effects of graphene-functionalized titanium carbide (Ti_3_C_2_) nanocomposites on cancer cell lines. The Ti_3_C_2_-graphene heterostructure was prepared by a simple cost-effective, hydrothermal method. The prepared Ti_3_C_2_-graphene material was characterized by various analytical techniques such as XRD, FE and SEM, and EDAX analysis. XRD analysis confirms the corresponding crystalline phases of Ti_3_C_2_ and graphene with a high crystalline nature. FE and SEM analysis shows Ti_3_C_2_ and graphene sheet-like morphology. The surface of the Ti_3_C_2_ sheets is modified by graphene and a large number of gaps (active sites). The study concludes that graphene is effectively interconnected with the MXene sheets, creating a new biocompatible yet cytotoxic material with a significant impact on cancer cell lines. The MTT assay test confirmed this cytotoxic behavior of Ti_3_C_2_-graphene heterostructure. The purity of the material was validated through XRD and EDS analyses. Further research is needed to confirm the biocompatibility of the synthesized material and its efficacy when combined with other substances to develop an effective cancer treatment. Additionally, the cytotoxic effects of graphene-functionalized Ti_3_C_2_ nanocomposites on cancer cell lines were investigated. Graphene is responsible for this biocompatibility because it enhances the dispersibility and stability of Ti_3_C_2_ nanoparticles in biological settings and offers a surface that is both biocompatible and bioinert for cell attachment and proliferation. To further strengthen their applicability for biomedical applications, the nanocomposites showed good colloidal stability and low aggregation in a cell culture medium.
